# Recent Advances in Doping and Polymer Hybridization Strategies for Enhancing ZnO-Based Gas Sensors

**DOI:** 10.3390/nano15211609

**Published:** 2025-10-22

**Authors:** Nazir Mustapha, Boutheina Ben Abdelaziz, Majdi Benamara, Mokhtar Hjiri

**Affiliations:** 1 Department of Physics, College of Sciences, Imam Mohammad Ibn Saud Islamic University (IMSIU), Riyadh 11623, Saudi Arabia; nmmustapha@imamu.edu.sa (N.M.); mbhjiri@imamu.edu.sa (M.H.); 2 Advanced Materials and Quantum Phenomena Laboratory, Physics Department, Faculty of Sciences of Tunis, Tunis El-Manar University, 2092 University Campus, Tunis 1006, Tunisia; bouthaina.benabdelaziz@insat.ucar.tn; 3 Physics Center of Minho and Porto Universities (CF-UM-UP), University of Minho, Campus de Gualtar, 4710-057 Braga, Portugal

**Keywords:** zinc oxide (ZnO), gas sensors, nanocomposites, dopant engineering, polymer functionalization, sol–gel synthesis, oxygen vacancies, room-temperature sensing, hybrid nanostructures, selectivity enhancement

## Abstract

Zinc oxide (ZnO) nanomaterials have emerged as promising candidates for gas sensing applications due to their high sensitivity, fast response–recovery cycles, thermal and chemical stability, and low fabrication cost. However, the performance of pristine ZnO remains limited by high operating temperatures, poor selectivity, and suboptimal detection at low gas concentrations. To address these limitations, significant research efforts have focused on dopant incorporation and polymer hybridization. This review summarizes recent advances in dopant engineering using elements such as Al, Ga, Mg, In, Sn, and transition metals (Co, Ni, Cu), which modulate ZnO’s crystal structure, defect density, carrier concentration, and surface activity—resulting in enhanced gas adsorption and electron transport. Furthermore, ZnO–polymer nanocomposites (e.g., with polyaniline, polypyrrole, PEG, and chitosan) exhibit improved flexibility, surface functionality, and room-temperature responsiveness due to the presence of active functional groups and tunable porosity. The synergistic combination of dopants and polymers facilitates enhanced charge transfer, increased surface area, and stronger gas–molecule interactions. Where applicable, sol–gel-based studies are explicitly highlighted and contrasted with non-sol–gel routes to show how synthesis controls defect chemistry, morphology, and sensing metrics. This review provides a comprehensive understanding of the structure–function relationships in doped ZnO and ZnO–polymer hybrids and offers guidelines for the rational design of next-generation, low-power, and selective gas sensors for environmental and industrial applications.

## 1. Introduction

Materials science translates atomic- and nano-scale structures and chemistry into macroscopic function across energy, information, health, and environmental resilience, with recent exemplars ranging from isotope-specific solvation guiding Zn-ion battery design and nanoconfined/porous architectures for catalysis and CO_2_ capture [1,2,3] to photonics/electronics, including upconversion photodynamic therapy, high-resolution printing of 3D curved electronics, and mid-IR hollow-core fibers [4,5,6]. Thermal management and extreme-environment metrology are being reshaped by heatsink-integrated 5G radomes, UV-DIC strain mapping to 3000 °C, and optimized heat removal in spindle systems [7,8,9,10]; advances in synthesis/joining and functional sorbents/absorbers—crystalline yttrium carbonate, MoS_2_/carbon composites, ultrasonic Cu/Cu, and DES-enabled sorbents—deliver application-tuned properties [11,12,13], increasingly co-designed with data-centric methods for perception, fusion, and decision-making [14,15,16,17,18] and with nano-bio platforms for cardiac, oncologic, and anti-infective applications [19,20,21,22,23,24]. At infrastructure scale, power-electronics control, vehicular platooning, and 6G edge offloading intersect with smart materials to stabilize cyber-physical networks [25,26,27,28,29,30,31], while systems optimization and autonomy (airport slots, on-orbit reconfiguration) set application pull [32,33]. Translational soft-matter/colloid control [34,35], secure blockchain-enabled mobility/logistics with multimodal learning [36,37], climate/process and resource-extraction interfaces [38,39,40], bio-interfaces and population-scale evidence [41], human–machine factors and resilient markets [42,43], and ESG-driven decarbonization [44] together sharpen materials targets alongside natural-product scaffolds and architected MoS_2_/carbon for microwave/photonic/catalytic response [45,46]. Ocean/subsurface monitoring, high-bandwidth power electronics, and mechatronic braking demand corrosion-resistant housings, high-κ/low-loss dielectrics, and wear-robust tribolayers [47,48,49,50]; environmental stressors, biomass hydrogenation, and propulsion stability further constrain chemistries and microstructures [51,52,53]; and rare-earth leaching kinetics, sonodynamic anti-infectives, weak-supervision perception, and learning-guided grids highlight the co-evolution of data and interfaces [54,55,56,57]. Innate-immunity mechanisms and policy-level carbon pathways frame adoption [58,59], while bio-inspired repair, computational imaging of transparent matter, agri-analytics, and medical vision press for biocompatible, optically clear, domain-adapted nanostructures [60,61,62,63]; finally, edge-deployable tensor frameworks, single-cell biomarkers, cavitation hydrodynamics, scene-level 3D occupancy, and steelmaking–casting scheduling close the loop from data to deployment [64,65,66,67,68]. Within this landscape, nanostructured oxides, especially ZnO, offer a tractable platform where dopant chemistry, defect engineering, and polymer hybridization tune carrier density, band alignment, and sorption kinetics to enable selective, fast, low-temperature gas sensing.

The continuous monitoring and detection of hazardous and environmentally relevant gases have become increasingly vital in today’s world due to growing industrialization, urbanization, and climate concerns [69,70,71]. Accurate and timely gas detection is critical not only for environmental monitoring and air quality control but also for industrial process safety, automotive emission management, agricultural productivity, and even healthcare diagnostics [72,73]. For instance, carbon monoxide (CO) is a colorless, odorless, and highly toxic gas that can accumulate in enclosed spaces due to incomplete combustion of fuels. Its early detection is essential to prevent fatal poisoning incidents, particularly in residential and workplace settings [74].

Similarly, hydrogen (H_2_) although widely used as a clean energy carrier in fuel cells and the chemical industry is extremely flammable and explosive even at low concentrations. Leaks must be rapidly detected to avoid catastrophic failures in storage and transportation infrastructure [75,76,77]. Methane (CH_4_), the primary component of natural gas, poses a dual threat: it is not only highly flammable but also a potent greenhouse gas with a global warming potential over 25 times higher than CO_2_ over a 100-year period [78,79,80]. Detecting CH_4_ emissions from pipelines, refineries, and landfills is thus critical for both safety and climate protection.

In the agricultural and medical sectors, ammonia (NH_3_) detection plays a key role. NH_3_ is a byproduct of livestock farming and fertilizer application and contributes significantly to air and water pollution [81]. It is also toxic at high levels and can irritate the respiratory system. In healthcare, exhaled ammonia levels serve as a non-invasive biomarker for kidney and liver dysfunction, offering potential for breath-based diagnostics [82,83,84]. Meanwhile, nitrogen dioxide (NO_2_), a toxic air pollutant produced by vehicle emissions and industrial combustion, can trigger asthma and other respiratory conditions at very low concentrations, necessitating high-sensitivity detectors in urban and indoor air monitoring systems [85,86,87].

Furthermore, the detection of volatile organic compounds (VOCs) such as ethanol, methanol, acetone, and formaldehyde (HCHO) has gained increasing importance due to their widespread use and associated health risks [88,89]. These VOCs are emitted from various industrial processes, consumer products, and biological sources. For example, ethanol detection is vital in breath analysis for law enforcement and medical diagnostics; methanol is highly toxic and used in solvents and antifreeze; acetone serves as a solvent in industries and as a biomarker in diabetes monitoring through breath analysis [90,91,92]. Formaldehyde, classified as a human carcinogen, is released from building materials, furniture, textiles, and disinfectants, posing serious health risks including respiratory irritation and cancer [93,94]. Its detection is crucial in indoor air quality monitoring, particularly in residential, educational, and occupational environments [95,96].

In response to these diverse application demands, metal oxide semiconductor (MOS) gas sensors have been widely studied and developed, owing to their simple operation, cost-effectiveness, and suitability for miniaturized and integrated systems [97]. Among the various MOS materials, zinc oxide (ZnO) stands out due to its wide bandgap (~3.37 eV), high electron mobility, thermal and chemical stability, and ease of synthesis in various nanoforms [98,99,100,101]. ZnO’s gas sensing mechanism typically involves adsorption of oxygen species (O_2_^−^, O^−^, O^2−^) on its surface, which capture electrons from the conduction band, creating a depletion layer [102,103,104,105]. Upon exposure to a reducing gas (e.g., H_2_, CO, NH_3_, VOCs), these surface species react with the target gas, releasing electrons back into the ZnO, reducing the depletion layer, and lowering the resistance [106,107]. Conversely, oxidizing gases like NO_2_ increase electron withdrawal, enhancing resistance [108,109].

However, pristine ZnO sensors still face several challenges, including limited selectivity between gases with similar redox behavior, insufficient sensitivity at low gas concentrations, and dependence on elevated operating temperatures (typically 250–400 °C) for optimal reaction kinetics [110,111,112,113]. These drawbacks pose barriers to their deployment in wearable, portable, or low-power devices, and in environments where high temperatures may be impractical or dangerous [114,115,116].

To address these limitations, researchers have extensively explored dopant engineering—introducing various metal and non-metal elements into the ZnO lattice—to modulate its electronic, structural, and surface properties. Dopants can: Enhance surface reactivity by generating more oxygen vacancies, Tune bandgap energy and Fermi level to improve carrier mobility, Create active sites for selective adsorption of specific gas molecules. Dopants such as Al, Ga, In (group III), Mg, Ca (group II), and transition metals like Co, Ni, Cu, Mn, Fe have shown promising results in improving the gas sensing performance of ZnO [117,118,119]. The effects vary depending on dopant type, concentration, ionic radius mismatch, and valence state, which can distort the ZnO lattice, promote charge separation, and influence interaction with target gas molecules [120,121].

An additional and increasingly explored strategy to enhance ZnO’s sensing performance especially for low-temperature or room-temperature applications is the integration of functional polymers. Conductive polymers such as polyaniline (PANI) and polypyrrole (PPy), as well as insulating or bio-based polymers like polyethylene glycol (PEG) and chitosan, can: Modify surface wettability and gas permeability, Provide functional groups for gas-specific interactions, Improve mechanical flexibility for wearable sensor applications, Enable hybrid architectures that facilitate charge transfer at interfaces, even at ambient conditions [122,123,124].

Polymer–ZnO composites exhibit synergistic effects polymers improve selectivity and flexibility, while ZnO offers stability and sensitivity. In some cases, polymers can act as templates or dispersing agents during sol–gel synthesis, further enhancing morphology control and porous structures [125,126]. The sol–gel method, known for its simplicity, scalability, and excellent control over homogeneity, has proven especially effective for synthesizing both doped ZnO nanostructures and ZnO–polymer hybrids. It enables precise tuning of parameters like porosity, grain size, surface area, and defect concentration—all of which are crucial for gas sensing behavior [127,128]. Complementary to polymer hybrids, graphene/GO/rGO–ZnO composites offer high-mobility pathways and abundant p–n junction interfaces that can boost room-temperature response and selectivity. Amination of graphene has been shown to enable uniform, thermally stable anchoring/dispersion of ZnO nanoparticles and to tune the interfacial electronic structure, rationalizing enhanced chemiresistive performance [129,130]. In parallel with ZnO, other binary and complex oxide sensors are advancing rapidly. For example, ZnGa_2_O_4_:Er ceramics enable robust high-temperature CH_4_ sensing for combustion monitoring; PEO-derived ZnO coatings exhibit gas-sensitive luminescence; and AlN nanoparticles show F-center luminescence useful for oxygen sensing—together broadening oxide-based sensing modalities beyond conductometric response [131,132,133].

**Figure 1:**
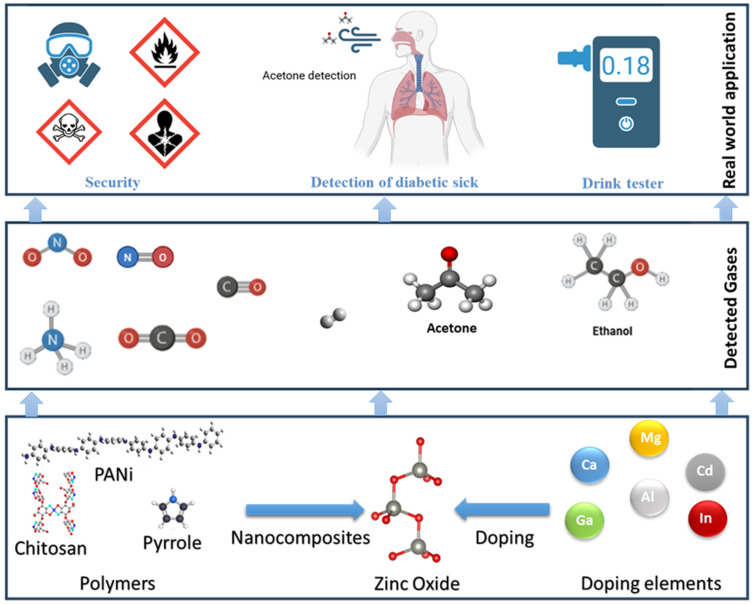
Doping and Polymer Integration Strategies for ZnO-Based Gas Sensors: From Material Design to Real-World Applications.

In this review, we critically assess and consolidate the recent progress in tailoring ZnO for gas sensing applications via dopant strategies and polymer integration, with a focus on materials synthesized through the sol–gel method. Special emphasis is placed on the mechanistic understanding of how these modifications enhance sensitivity, selectivity, and temperature adaptability for key target gases such as CO, H_2_, CH_4_, NH_3_, NO_2_, and VOCs including ethanol, methanol, acetone, and formaldehyde. This review covers multiple synthesis routes but emphasizes sol–gel-derived ZnO (including spin/dip-coating and gel-combustion variants). For clarity, each highlighted result is tagged by synthesis route and we summarize how sol–gel processing alters oxygen-vacancy chemistry, grain size/porosity, and sensor figures of merit. We also highlight real-world use cases and address future prospects in developing next-generation ZnO-based gas sensors for environmental, industrial, and biomedical applications (Figure 1).

## 2. ZnO Gas Sensing Mechanism

The gas sensing performance of ZnO is primarily governed by surface interactions with gas molecules, which modulate the electrical conductivity of the material. This section outlines the core mechanisms that define ZnO’s response to various gases, focusing on the roles of surface reactions, adsorbed oxygen species, operational temperature, and intrinsic or dopant-induced defects particularly oxygen vacancies [134,135].

**Figure 2:**
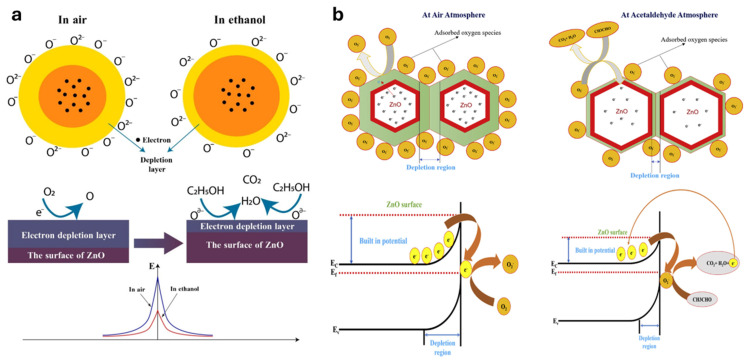
(**a**) Proposed reaction mechanism of ZnO toward ethanol, showing depletion-layer changes. Reproduced from [144]. (**b**) Proposed sensing mechanism of ZnO toward acetaldehyde (adsorbed oxygen, depletion modulation, grain-boundary transfer). Reproduced from [145] with permission.

As illustrated in Figure 2a, the conductometric sensing mechanism of ZnO-based sensors relies heavily on the modulation of the electron depletion layer due to surface interactions with oxygen and target gas molecules such as ethanol. In ambient air, oxygen molecules adsorb onto the ZnO surface and extract electrons from its conduction band, forming negatively charged oxygen species (O_2_^−^, O^−^, O^2−^) and creating an electron-depleted region near the surface [136,137,138]. This results in increased resistance. When exposed to ethanol, these molecules react with the chemisorbed oxygen, releasing electrons back into the conduction band, thus reducing the depletion layer and decreasing resistance—an effect that enhances conductivity. This dynamic modulation of resistance forms the basis of ZnO’s sensing response [139,140].

Figure 2b further exemplifies the sensing process in the context of acetaldehyde detection. Here, a schematic of adjacent ZnO grains shows oxygen species adsorbing and forming depletion regions at the grain boundaries in air. Upon exposure to acetaldehyde, a redox reaction occurs between the gas and surface oxygen species, producing CO_2_, H_2_O, and releasing electrons [141,142]. This electron injection diminishes the potential barrier at the ZnO grain interfaces, leading to a significant drop in resistance. The conduction band model in Figure 2b visually supports this by illustrating the shift in built-in potential and narrowing of the depletion region under the influence of the target gas. These examples collectively highlight how electron transfer processes at the gas–solid interface regulate the sensing behavior of ZnO nanostructures [143,144,145].

### 2.1. Surface Reactions with Gases

**Figure 3:**
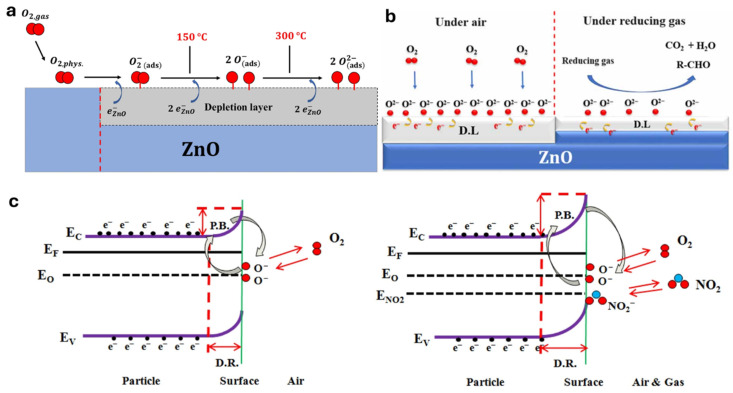
(**a**) Principle of oxygen adsorption on ZnO-sensor surfaces. Reproduced from [157]. (**b**) Gas-sensing process in air vs. reducing gas. Reproduced from [158]. (**c**) Gas-sensing process of ZnO in air vs. NO_2_ (oxidizing gas). Reproduced from [157]; all with permission.

The gas sensing behavior of ZnO, an intrinsic *n*-type semiconductor, is governed by surface adsorption and desorption processes that modulate its conduction-band electron density. During crystal growth, intrinsic oxygen vacancies (V_O_) act as donor levels, releasing electrons into the conduction band and serving as preferential adsorption sites for oxygen molecules from the ambient air [146,147,148]. When O_2_ encounters the ZnO surface, it physisorbs and then ionizes by capturing electrons, forming a sequence of oxygen species whose nature depends on temperature: at low temperatures (≤150 °C), O_2_ + e^−^ → O_2_^−^; at moderate temperatures (150–300 °C), O_2_^−^ + e^−^ → 2 O^−^; and at high temperatures (>300 °C), O^−^ + e^−^ → O^2−^ (Figure 3a). These ionized species create a depletion layer by extracting electrons from the conduction band, which raises the surface potential barrier and causes an increase in electrical resistance [149,150].

The selection of operating temperature thus represents a trade-off between sensitivity, selectivity, and power consumption. Below 150 °C, the weakly reactive O_2_^−^ species limit sensitivity and slow kinetics. Between 150 and 300 °C, the formation of highly reactive O^−^ species enhances gas–surface interactions and accelerates sensor dynamics. Above 300 °C, although O^2−^ species dominate, increased desorption of adsorbed ions reduces overall sensor response, necessitating careful optimization to achieve the best performance [151,152,153].

When the ZnO sensor is exposed to reducing gases such as H_2_, CO, CH_4_, ethanol, or formaldehyde, these gases react with the adsorbed oxygen ions and release trapped electrons back into the conduction band. For instance, H_2_ + O^−^ → H_2_O + e^−^, CO + O^−^ → CO_2_ + e^−^, and C_2_H_5_OH + O_2_^−^ → CH_3_CHO + H_2_O + 2 e^−^. This electron reinjection narrows the depletion layer and lowers resistance, producing a pronounced change in conductivity [154,155]. Experimental resistance transients under moist air and various VOC concentrations at 300 °C demonstrate that ionosorption of oxygen—and thus sensitivity—is maximized at this operating temperature, where response and recovery are also fastest (Figure 3b).

Oxidizing gases such as NO_2_, by contrast, increase ZnO resistance through additional electron withdrawal. NO_2_ molecules adsorb onto the surface and capture electrons more readily than oxygen (Ea ≈ 2.04 eV versus 0.48 eV), forming NO_2_^−^ (NO_2_ + e^−^ → NO_2_^−^) and subsequently reacting with O^−^ to yield NO and O^2−^ (NO_2_^−^ + O^−^ + 2 e^−^ → NO + 2 O^2−^) [156,157,158]. This deepens the depletion region and raises the potential barrier even further, causing a significant resistance increase (Figure 3c). The higher activation energy and slow desorption kinetics of NO_2_ result in a slower, less reversible sensor response compared to reducing gases.

### 2.2. Role of Defects and Polymer Synergy in ZnO Gas Sensing Mechanisms

Defects, particularly oxygen vacancies (V_o_), and the incorporation of dopants play a critical role in enhancing the gas sensing behavior of ZnO-based materials. Oxygen vacancies serve as essential active sites for oxygen adsorption and subsequent gas interactions, functioning as electron donors that enhance the n-type conductivity and increase the density of reactive surface sites [159,160]. These vacancies also promote gas diffusion and catalyze redox reactions on the sensor surface. Through sol–gel synthesis, it is possible to precisely control the concentration of such defects, a process further refined by strategic dopant engineering. Different dopants influence vacancy formation and alter the band structure and Fermi level of ZnO in unique ways. For instance, Group III elements like Al, Ga, and In increase the free electron concentration, reduce grain size, and promote the formation of oxygen vacancies. Transition metal dopants such as Co, Cu, and Ni introduce localized states that facilitate charge transfer and modulate interactions with gas molecules, while Group II dopants like Mg and Ca affect lattice distortion and surface polarity, thus fine-tuning adsorption and desorption kinetics. These modifications not only improve sensitivity and reactivity but also enhance selectivity by creating favorable interaction sites for specific gases [161,162].

Beyond inorganic modification, the integration of polymers into ZnO-based sensors introduces an additional level of functional versatility. In polymer–ZnO hybrid systems, the sensing mechanism transcends the conventional behavior of metal oxides. Polymers contribute functional groups—such as –NH_2_ in chitosan or –COOH in PEG—that provide selective interaction sites for specific gas molecules, greatly enhancing the sensor’s selectivity [163,164]. These organic components also support room-temperature sensing by adjusting surface charge distribution and forming heterojunctions that promote efficient interfacial charge transfer even under ambient conditions. For example, in ZnO–PANI hybrids, exposure to NH_3_ triggers the deprotonation of PANI, which alters its conductivity and modifies the interfacial potential barrier with ZnO. Similarly, in ZnO–chitosan systems, the polymer matrix not only improves gas permeability and sensor stability but also sustains effective sensing performance at lower temperatures. Together, the synergistic roles of defects, dopants, and polymers contribute to the design of highly responsive, selective, and energy-efficient gas sensing materials [165,166].

## 3. Fabrication and Characterization of ZnO-Based Gas Sensors

### 3.1. Sol–Gel Synthesis of ZnO Nanostructures for Gas Sensing

In the sections that follow, we distinguish sol–gel (SG) and non-sol–gel (NSG) studies to isolate synthesis-route effects on defect density, microstructure, and performance.

The sol–gel technique is a solution-based synthesis method widely used for the preparation of metal oxide materials with controlled stoichiometry, tunable morphology, and compositional uniformity at relatively low processing temperatures. In the case of ZnO, the process typically begins with dissolving zinc precursors such as zinc acetate or zinc nitrate in solvents like ethanol or water [167,168]. Through hydrolysis and condensation reactions, a colloidal suspension (sol) forms, which gradually transitions into a gel-like network. Upon drying and thermal treatment, the gel converts into crystalline ZnO. The simplicity, cost-effectiveness, and versatility of the sol–gel method make it an ideal platform for the integration of functional components—including dopants and polymers—within the ZnO matrix [169,170].

An important advantage of the sol–gel method is its ability to control the morphology of the resulting ZnO nanostructures. By tuning synthesis parameters such as precursor concentration, solvent type, aging time, and calcination conditions, various architectures—ranging from nanoparticles and nanorods to nanosheets and mesoporous frameworks—can be obtained. Such morphological tailoring is critical for gas sensing applications because it directly affects surface area, pore structure, and grain boundary density [171,172]. Porous and nanostructured ZnO provides more surface-active sites for gas adsorption and facilitates faster diffusion pathways, leading to enhanced sensor sensitivity and quicker response times. Additionally, the introduction of polymers during sol–gel processing can further refine the material’s morphology. Polymers act as structure-directing agents or sacrificial templates, promoting the formation of porous networks while simultaneously imparting mechanical flexibility and improved film uniformity, which are especially beneficial for flexible and wearable gas sensor platforms.

The sol–gel approach also enables the precise incorporation of dopants into the ZnO crystal lattice. Dopant elements such as Al, Ga, Mg, Cu, Co, and Ni can be introduced during the sol stage, ensuring homogeneous distribution throughout the final material. These dopants can substitute Zn^2+^ ions or occupy interstitial positions, leading to lattice distortions and electronic structure modifications. As a result, doped ZnO exhibits improved carrier mobility, increased oxygen vacancy concentrations, and adjusted bandgap energies all factors that significantly enhance gas sensing characteristics. Transition metal dopants, in particular, introduce localized electronic states that facilitate charge transfer interactions with adsorbed gas species, thereby boosting both sensitivity and selectivity.

Moreover, combining ZnO with polymers through sol–gel processing creates hybrid nanocomposites that synergize the advantageous properties of both components. The polymer matrix can enhance gas selectivity by acting as a molecular sieve or provide a flexible support network for ZnO nanoparticles, thus improving adhesion, stability, and mechanical robustness of the sensing layer. Such polymer–ZnO composites are increasingly attractive for next-generation gas sensors, where high sensitivity, mechanical flexibility, and device miniaturization are critical requirements [173,174].

### 3.2. Gas Sensor Instrumentation and Measurement Methodology

Semiconducting metal oxide-based gas sensors detect target gases through changes in the electrical resistance of their sensing layers. In air, oxygen molecules adsorb on the surface and capture free electrons, forming an electron depletion layer that increases resistance. When reducing or oxidizing gases are introduced, surface reactions release or consume electrons, causing a measurable resistance change—the fundamental principle of resistive gas sensing [175,176].

Typically, sensors are tested in chambers where gas composition and flow are controlled using mass flow controllers and valves. The sensing material, deposited on a substrate with interdigitated electrodes and a heating element, is connected to a data acquisition system for real-time resistance monitoring. The heater maintains the optimal operating temperature for surface reactions. The gas response (S) is defined as S = R_a_/R_g_ for reducing gases, where *R_a_* and *Rg* are the resistances in air and in the target gas, respectively [177,178].

**Figure 4:**
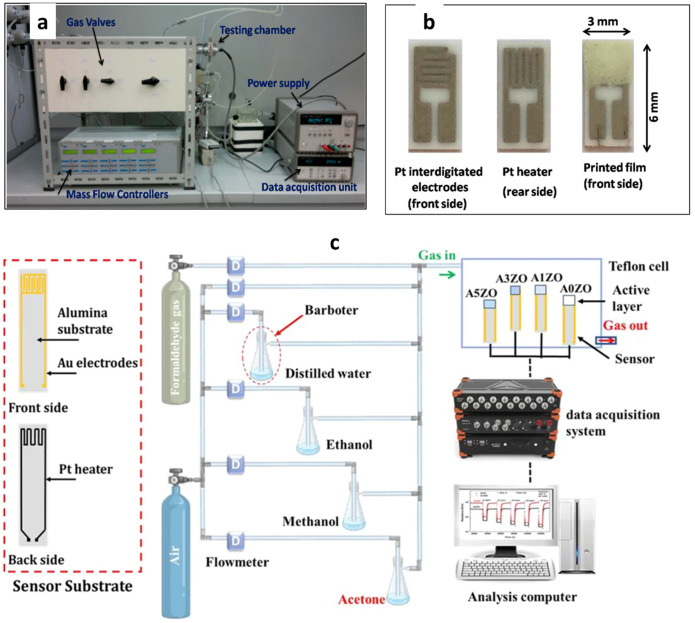
Experimental setup and devices. (**a**) Gas-sensing experimental setup. Reproduced from with permission. (**b**) Sensor components and device structure (Pt heater, Pt IDEs, printed film). Reproduced from [181] with permission. (**c**) sensor substrate and Sensing device [182].

Gas sensing followed standard conductometric practice (Figure 4a,b). ZnO-based films were deposited on substrates with interdigitated electrodes and integrated heaters, then tested in sealed chambers under controlled flow and temperature. Resistance was continuously recorded, and responses were calculated as S = R_a_/R_g_ for reducing or R_g_/R_a_ for oxidizing gases. The baseline resistance of pristine and doped films was also measured versus temperature. At low temperatures, high resistance resulted from carrier freeze-out and ionized impurity scattering; with increasing temperature, carrier activation and mobility increased, reducing resistance. Doped ZnO films showed a stronger resistance decrease with temperature, indicating improved carrier transport and enhanced sensing due to dopant-induced states [179,180,181].

Gas-sensing tests were performed using a custom-built system (Figure 4c) comprising a Teflon chamber connected to a data acquisition unit for resistance measurements under controlled conditions. Two gas cylinders (formaldehyde and air) and four bubblers containing distilled water and dilute ethanol, methanol, and acetone solutions provided humidity and target-gas concentrations. Before testing, synthetic air (79% N_2_ + 21% O_2_) was injected until baseline resistance stabilized. Acetone vapor was then introduced at 5–50 ppm for 15 min, followed by chamber evacuation. Tests were performed at 50% relative humidity by bubbling gas through distilled water. The gas response was calculated as R = (R_air_ − R_gas_)/R_gas_, and response/recovery times corresponded to 90% of total resistance change during exposure and re-exposure.

To ensure stability at high temperatures, sensors were operated between 200 and 350 °C while avoiding direct contact with the Teflon walls. The chamber temperature was monitored and cooled when needed to prevent Teflon degradation, ensuring reliable and reproducible measurements.

## 4. Dopant Strategies

Doping ZnO with cationic and transition metal elements is a widely adopted strategy to tailor its electronic, structural, and surface characteristics for enhanced gas sensing performance. Transition metals such as Cu, Co, Ni, Mn, and Fe are particularly effective due to their ability to introduce localized electronic states and modulate charge carrier dynamics. These dopants typically substitute Zn^2+^ ions in the lattice, causing slight distortions due to ionic radius mismatch, which leads to the formation of additional oxygen vacancies key active sites for gas adsorption and redox reactions [183,184]. For example, Cu-doped ZnO has shown improved sensitivity to reducing gases like CO and H_2_ due to enhanced electron mobility and a higher density of surface-active sites. Similarly, Al or Ga doping (from group III elements) contributes to an increase in free electron concentration by acting as shallow donors, thus improving conductivity and response times. The type and concentration of dopants play a critical role; low concentrations typically improve sensing by optimizing the balance between conductivity and defect density, while excessive doping can lead to secondary phase formation or defect saturation, negatively impacting performance. The sol–gel method, by virtue of its atomic-level mixing and low-temperature processing, ensures homogeneous dopant distribution and minimizes the risk of unwanted agglomeration or phase segregation [185,186].

Rare-earth elements such as gadolinium (Gd), lanthanum (La), cerium (Ce), and others are also gaining attention for their unique role in enhancing gas sensor functionalities. These dopants not only introduce localized energy states near the conduction band but also exhibit high oxygen affinity, which supports the formation and stabilization of surface oxygen species crucial for gas-sensing reactions. For instance, Gd-doped ZnO exhibits improved selectivity and lower detection limits for gases like NO_2_ and ethanol, attributed to its strong interaction with oxygen and its ability to modulate charge carrier density effectively [187,188]. The large ionic radii of rare-earth ions often induce significant lattice distortions and strain, which in turn create additional active sites and increase surface roughness—factors that promote greater gas adsorption. Furthermore, rare-earth dopants can influence the formation of heterojunctions and interfacial band alignment when used in composite systems, particularly with polymers or other metal oxides. These effects not only enhance sensitivity but also allow for improved operation at lower temperatures, making rare-earth-doped ZnO especially suitable for wearable and portable sensor platforms. Overall, both transition metal and rare-earth doping approaches offer complementary pathways to engineer ZnO at the atomic level, opening new avenues for designing high-performance gas sensors with precise control over sensitivity, selectivity, and stability [189,190].

### 4.1. Doping Effect on the Sensitivity

**Figure 5:**
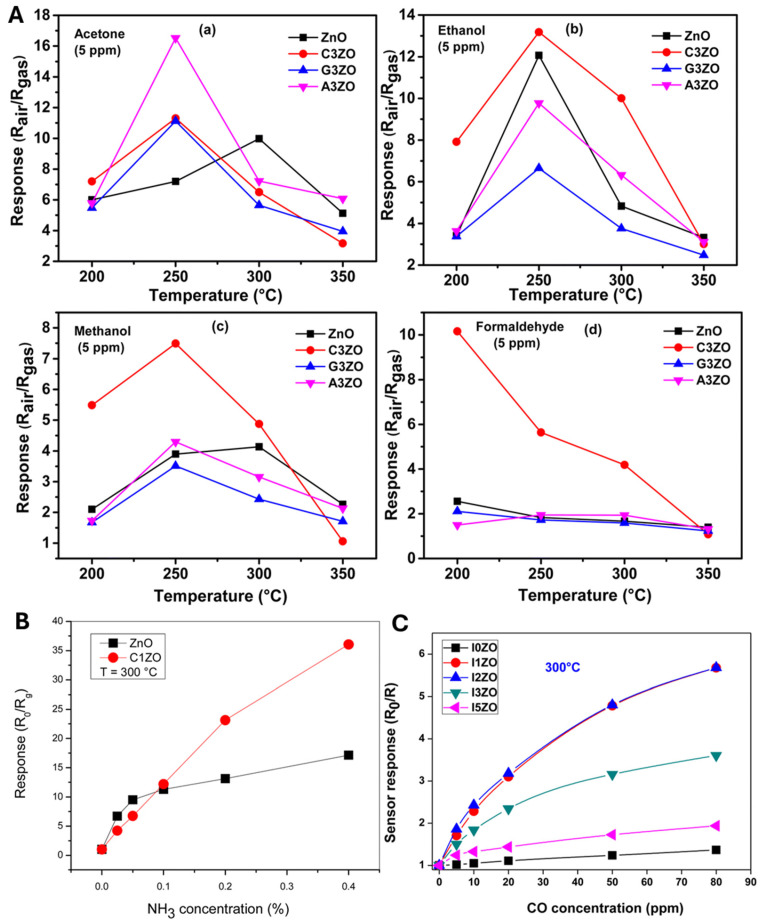
Representative sensor responses. (**A**) Responses of ZnO, C3ZO, G3ZO, and A3ZO to 5 ppm acetone (**a**)/ethanol (**b**)/methanol (**c**)/formaldehyde (**d**) at various temperatures. Reproduced from [191] with permission. (**B**) NH_3_ responses of pure vs. 1% Ca-doped ZnO at 300 °C. Reproduced from [192]. (**C**) CO sensing of pure vs. In-doped ZnO at 300 °C. Reproduced from [193] with permission.

The prepared sensors were tested in the presence of 5 ppm of acetone, ethanol, methanol, and formaldehyde gases with 50% RH at different temperatures ranging from 200 to 350 °C. The sensor responses to VOC gases are shown in Figure 5A(a–d). The curves indicate that the highest responses were achieved at around 250 °C. The doped sensors exhibited superior responses compared to the pure ZnO sensor. In particular, the A3ZO (Al_3%_ doped ZnO) sensor showed the best response to acetone gas, while the C3ZO (Ca_3%_ doped ZnO) sensor demonstrated the highest responses for ethanol, methanol, and formaldehyde. The enhanced performance upon doping is mainly attributed to the creation of structural defects, such as oxygen vacancies and interstitials, which play crucial roles in gas adsorption and reaction mechanisms. Specifically, the improved acetone sensing performance of the A3ZO sensor could be ascribed to its smaller particle size and higher specific surface area, promoting easier gas diffusion and stronger interaction with adsorbed oxygen species. Moreover, a larger number of oxygen vacancies serve as active sites, significantly improving gas molecule adsorption and thus enhancing sensor response.

The gas response of pure and Ca-doped ZnO sensors to ammonia (NH_3_) at 300 °C is depicted in Figure 5B. At lower NH_3_ concentrations, pure ZnO shows a higher response, but as the concentration increases, the C1ZO sensor outperforms the undoped ZnO, achieving a maximum response of 33. The enhancement in response with Ca doping is linked to the generation of donor defects like oxygen vacancies and zinc interstitials, which increase the free electron concentration, facilitating better interaction with NH_3_ molecules. Furthermore, Ca ions help maintain a cleaner surface, promoting faster desorption and recovery compared to the pure ZnO sensor.

The calibration curves for In-doped ZnO (IZO) sensors for CO detection at 300 °C are presented in Figure 5C. The sensor response increases with CO concentration and peaks at 1–2 at.% In doping. Beyond this optimal doping, the response declines due to potential grain agglomeration, which reduces the effective surface area. The enhanced response at low In doping levels is associated with an increased number of active sites for oxygen species adsorption and better charge carrier liberation during CO exposure.

### 4.2. Doping Effects on the Response and Recovery Dynamics of ZnO-Based Gas Sensors

The response and recovery times of gas sensors are critical parameters for evaluating their practical performance, as they determine how rapidly a sensor can detect and release target gas molecules. In the case of ZnO-based sensors, doping with rare-earth elements and transition metals has been widely employed to modulate the sensing kinetics. These dopants influence the density of surface-active sites, modify the carrier concentration, and tailor the oxygen adsorption–desorption processes, thereby impacting the rate at which gases interact with and detach from the sensing surface. Faster response and recovery dynamics are generally desirable for applications requiring real-time gas monitoring and rapid detection [194,195].

**Figure 6:**
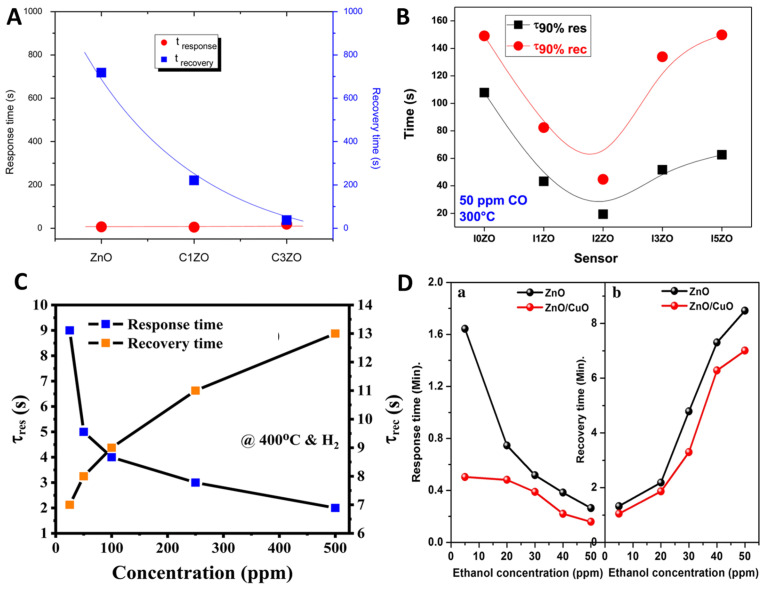
Response and recovery metrics. (**A**) Ca-doped ZnO response/recovery times. Reproduced from [191]. (**B**) In-doped ZnO response/recovery at 300 °C. Reproduced from [193]. (**C**) Response and recovery times of the Ag/Pd (0.025 wt%)-doped ZnO nanoplate sensor as a function of H_2_ concentration at 400 °C. Reproduced from [106] (**D**) Response (**a**) and recovery (**b**) times of ZnO and ZnO/CuO sensors toward ethanol gas. Reproduced from [196] with permission.

Figure 6 presents representative examples illustrating the impact of various dopants on the response and recovery times of ZnO gas sensors. In Figure 6A, the response and recovery behaviors of pure ZnO and Ca-doped ZnO samples (C1ZO and C3ZO) are compared. Although all samples exhibit rapid response times (6 s for ZnO, 5 s for C1ZO, and 18 s for C3ZO), the recovery times show notable differences, with pure ZnO and C1ZO demonstrating much slower recovery (718 s and 221 s, respectively) compared to the relatively faster recovery of C3ZO (37 s). This behavior suggests that doping with Ca improves the desorption kinetics of adsorbed ammonia species, likely by modifying the surface energy landscape and promoting weaker binding between gas molecules and the sensing layer.

In Figure 6B, the effect of indium (In) doping on ZnO sensor dynamics is depicted. The incorporation of In leads to a marked improvement in the response and recovery characteristics towards CO gas. Low-level In doping (1–2 at.%) enhances the accessibility and reactivity of surface sites, resulting in faster signal stabilization after exposure to CO. However, at higher doping concentrations (3–5 at.%), a slight deterioration in the response/recovery times is observed, likely due to excessive lattice distortion or the formation of localized trap states. These findings highlight that an optimal dopant concentration is critical to balancing surface reactivity and carrier transport for efficient gas sensing.

The response and recovery times of the sensors were determined from the transient response curves, defined as the time required to reach 90% of the total resistance change upon gas exposure and re-exposure. As shown in Figure 6C, both parameters provide valuable insight into adsorption–desorption kinetics. For example, the Ag/Pd(0.025 wt%)-doped ZnO nanoplate sensor exhibited ultrafast dynamics at 400 °C, with response and recovery times of approximately 2 s and 13 s, respectively, for 500 ppm H_2_ gas. The response time increased slightly with decreasing gas concentration, whereas the recovery time tended to shorten under lower H_2_ concentrations. This inverse behavior is attributed to the adsorption–desorption mechanism: at high gas concentrations, target molecules readily interact with the surface, accelerating resistance changes; at lower concentrations, desorption from the surface becomes easier once the environment is refreshed by airflow.

A similar trend was observed for ethanol detection, as illustrated in Figure 6D. The response time decreased with increasing gas concentration, while the recovery time increased correspondingly. This is because, at higher concentrations, a greater number of gas molecules possess the minimum activation energy required for reaction, resulting in faster resistance change. Conversely, at lower concentrations, reduced surface coverage leads to slower adsorption and delayed sensor response. Remarkably, both pristine ZnO and ZnO/CuO sensors demonstrated efficient ethanol sensing even at room temperature, responding to 5 ppm within less than 100 s. The calculated response times were 98 s for ZnO and 30 s for ZnO/CuO, with nearly complete desorption achieved within a few minutes, particularly at low concentrations.

Overall, these examples clearly demonstrate that careful selection and optimization of dopant type and concentration significantly influence the response and recovery behavior of ZnO-based gas sensors, offering valuable strategies to enhance sensing rapidity for different target gases.

### 4.3. Effect of Doping Elements on the Reproducibility of ZnO-Based Gas Sensors

Reproducibility is a critical parameter in gas sensor evaluation, as it reflects the sensor’s ability to deliver consistent and stable responses over multiple gas exposure cycles under identical experimental conditions. In ZnO-based gas sensors, reproducibility directly impacts reliability, operational stability, and suitability for practical applications. Several factors can affect reproducibility, including fluctuations in surface reactivity, instability of charge carrier concentrations, and material degradation over time. Doping ZnO with suitable foreign elements has been demonstrated to significantly enhance reproducibility by modifying the intrinsic defect structure, improving the chemical and thermal stability of the material, and stabilizing the adsorption–desorption dynamics of target gases at the sensor surface [197,198]. Dopants can introduce new energy levels within the bandgap, regulate the concentration of oxygen vacancies, and control grain boundary characteristics, leading to more uniform charge transport and surface reactions during repeated sensing cycles. Consequently, doped ZnO sensors typically exhibit minimal baseline drift and maintain highly repeatable response and recovery behaviors across successive gas exposure and purging cycles. The improvement in reproducibility through doping strategies is crucial for ensuring reliable long-term sensor operation, particularly in real-world environments where fluctuations in temperature, humidity, and gas concentration are inevitable [199,200].

**Figure 7:**
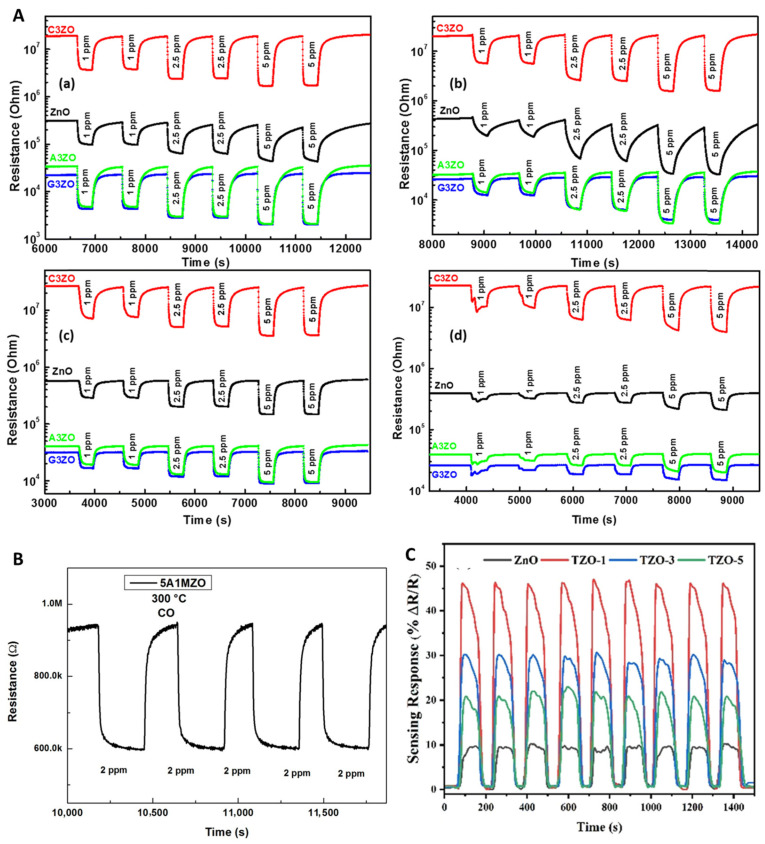
(**A**) Time-dependent resistance of ZnO, C3ZO, A3ZO, and G3ZO to (**a**) acetone, (**b**) ethanol, (**c**) methanol, and (**d**) formaldehyde gases at 250 °C, 50% RH (two repeats per concentration). Reproduced from [191]. Reproducibility and repeatability. (**B**) Dynamic resistance reproducibility of Al_5%_–Mg_1%_ co-doped ZnO toward H_2_. Reproduced from [181]. (**C**) Repeatability of ZnO and Ti-doped ZnO sensors. Reproduced from [201]. All references are with permission.

These results underline the reliability of ZnO-based sensors enhanced by co-doping strategies. The effect of individual dopants such as Ca, Al, and Ga was further investigated through dynamic resistance measurements under exposure to acetone, ethanol, methanol, and formaldehyde gases (Figure 7A(a–d)). For instance, the Al_5_%-Mg_1_% co-doped ZnO sensor (5A1MZO) exhibited stable and repeatable resistance responses over four consecutive cycles during exposure to 20 ppm CO at 300 °C, as shown in Figure 7B.

ZnO, Ca_3_%-doped ZnO (C_3_ZO), Al_3_%-doped ZnO (A_3_ZO), and Ga_3_%-doped ZnO (G_3_ZO) sensors were tested at low gas concentrations (1, 2.5, and 5 ppm) under 50% RH at 250 °C. Across two consecutive injections for each concentration, the sensors showed almost identical responses, confirming the significant enhancement of reproducibility at low VOC concentrations through appropriate doping. In addition, Ti-doped ZnO sensors demonstrated excellent repeatability in response to NO gas at 220 °C, with nine consecutive cycles showing negligible variation in sensing behavior (Figure 7C). This highlights the robustness and durability of Ti incorporation in the ZnO matrix.

### 4.4. Effect of Doping Elements on the Selectivity Behavior of ZnO Gas Sensors

Selectivity, defined as the ability of a gas sensor to distinguish between different gaseous species, is a crucial parameter for ensuring reliable performance in complex environments. High selectivity minimizes cross-sensitivity issues that often compromise measurement accuracy and repeatability, particularly in practical applications involving multiple interfering gases. To enhance selectivity, strategies such as doping with transition metals and rare-earth elements have been widely explored. These dopants modify the electronic structure, surface chemistry, and adsorption–desorption dynamics of ZnO, leading to improved discrimination against non-target gases [202,203]. Various examples highlight how doped ZnO systems respond preferentially to specific target gases under different operating conditions.

**Figure 8:**
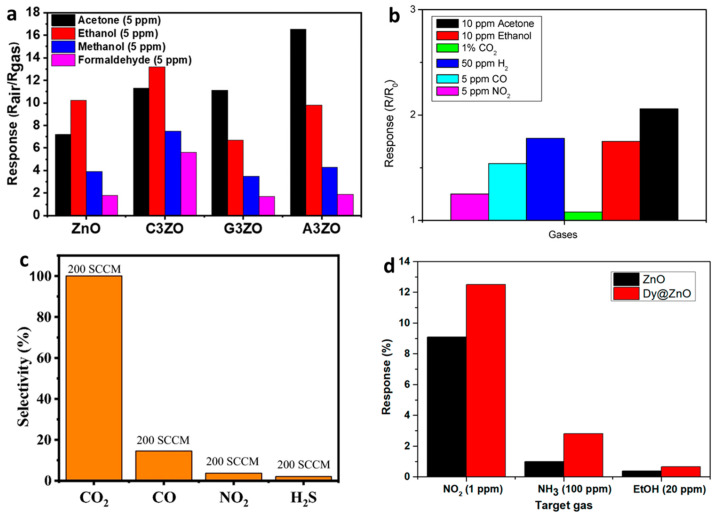
Selectivity comparisons. (**a**) Responses of ZnO, C3ZO, A3ZO, and G3ZO to 5 ppm target gases at 250 °C, 50% RH. Reproduced from [191]. (**b**) Co-doped ZnO selectivity to H_2_, acetone, ethanol, and interferents. Reproduced from [204]. (**c**) Selectivity of 4.0 at% La-doped ZnO at room temperature, 30% RH. Reproduced from [205]. (**d**) Comparative responses of Dy-doped vs. pure ZnO. Reproduced from [206]. All references are with permission.

For instance, Ca-, Ga-, and Al-doped ZnO (denoted as C3ZO, G3ZO, and A3ZO, respectively) sensors exhibited differentiated selectivity patterns when exposed to 5 ppm of acetone, ethanol, methanol, and formaldehyde at 250 °C and 50% RH (Figure 8a). Pure ZnO showed higher sensitivity towards ethanol; however, upon Ca doping, the C3ZO sensor maintained strong selectivity toward ethanol, slightly surpassing that for acetone. Notably, Al doping significantly enhanced the response toward acetone, suggesting that Al modifies the ZnO surface to favor acetone adsorption. This enhanced selectivity is attributed to the differences in the dipole moments of the target gases—acetone (2.91 D) having a higher value compared to ethanol (1.66 D), methanol (1.70 D), and formaldehyde (2.33 D)—as well as factors like molecular size and weight.

Similarly, Co-doped ZnO demonstrated improved selective behavior toward hydrogen (H_2_) over other gases like acetone and ethanol (Figure 8b). The sensor showed a considerably higher response to H_2_, confirming the ability of Co doping to tailor ZnO’s electronic properties towards enhanced H_2_ sensing. Rare-earth doping strategies were equally effective [204]. For instance, ZnO doped with 4.0 at% La exhibited exceptional selectivity toward CO_2_ at room temperature and 30% RH (Figure 8c). The La-doped ZnO sensor showed a much higher response to CO_2_ (114.22%) compared to CO, NO_2_, and H_2_S, confirming La’s role in tuning ZnO’s adsorption preference towards CO_2_ [205]. Finally, Dy-doping provided ZnO with superior NO_2_ selectivity (Figure 8d). When comparing responses to 1 ppm NO_2_, 100 ppm NH_3_, and 20 ppm ethanol at 150 °C, Dy-doped ZnO showed significantly higher responses toward NO_2_, even at a lower concentration. This enhanced performance is supported by DFT calculations showing that NO_2_ molecules possess stronger binding and greater charge transfer interactions with ZnO surfaces, particularly when doped with Dy, leading to enhanced sensor dynamics and resistance changes. Across dopants surveyed, sol–gel-derived ZnO tended to show finer grains/greater porosity and higher active-oxygen density, correlating with higher responses at ≤250–300 °C versus non-sol–gel counterparts reporting similar chemistries [206].

## 5. Integration of Polymer Matrices

Another promising strategy enabled by sol–gel synthesis is the incorporation of polymers into ZnO-based structures. Both conductive and insulating polymers can be integrated to enhance sensor performance. Conductive polymers such as polyaniline (PANI) and polypyrrole (PPy), and insulating or bio-based polymers like polyethylene glycol (PEG) and chitosan, may be blended into the sol prior to gelation or used as structural templates. These polymers introduce functional groups capable of selectively interacting with gas molecules, improving gas permeability and the mechanical flexibility of the sensor films. Additionally, they influence the nucleation and growth of ZnO nanostructures, enabling better control over particle size, dispersion, and porosity [207,208].

The integration of conducting polymers into ZnO materials has proven particularly effective in addressing the limitations of pure metal oxide gas sensors, especially with regard to sensitivity, selectivity, and high-temperature operation. PANI and PPy are widely studied due to their good electrical conductivity, environmental stability, and ease of processing. When combined with ZnO nanostructures, they form hybrid composites that exhibit improved charge transport and strong interfacial interactions with gas molecules. This enhancement is largely due to the formation of p–n heterojunctions at the interface between the n-type ZnO and the p-type polymer. These junctions generate potential barriers that are modulated upon exposure to target gases, resulting in amplified resistance changes. As a result, such composites can operate efficiently even at room temperature. Moreover, the porous, flexible nature of the polymers facilitates rapid gas diffusion and adsorption, while their functional groups enable selective detection of specific gases. This makes conducting polymer/ZnO composites particularly effective for detecting gases like ammonia (NH_3_), hydrogen sulfide (H_2_S), and volatile organic compounds (VOCs) under ambient conditions without external heating.

In addition to conducting polymers, non-conductive natural and synthetic polymers such as chitosan, polyvinyl alcohol (PVA), and sodium alginate are also utilized to enhance ZnO-based gas sensors [209,210]. Although these materials lack intrinsic conductivity, they offer unique benefits including biocompatibility, excellent film-forming ability, and high surface area when processed as thin films or electrospun fibers. For example, chitosan provides amino and hydroxyl groups that can interact selectively with polar or acidic gases like formaldehyde and nitrogen dioxide (NO_2_), thereby improving adsorption kinetics and selectivity. Similarly, PVA and alginate can act as flexible templates that influence ZnO dispersion and support the formation of porous morphologies during sol–gel processing. These insulating polymers also contribute to mechanical robustness and flexibility, which are desirable features for wearable and flexible electronic applications. Furthermore, insulating polymers can serve as selective gas diffusion barriers, allowing specific molecules to reach the ZnO surface while limiting interference from others—thereby enhancing sensor selectivity. Overall, the use of both conductive and insulating polymer matrices in ZnO composites leads to multifunctional gas sensors that combine improved sensitivity, selectivity, and operational stability under low-temperature and low-power conditions [122,211].

### 5.1. Gas Sensing Behavior of PANi–ZnO Polymer Nanocomposites

Among polymer–metal oxide hybrids, polyaniline (PANi)–ZnO nanocomposites have gained considerable attention for gas sensing owing to their tunable electrical conductivity, high surface activity, and structural stability. The combination of the conducting polymer matrix and ZnO nanoparticles facilitates efficient charge transfer and enhances adsorption–desorption kinetics, making these materials highly responsive to various reducing and oxidizing gases under mild operating conditions. Several studies have highlighted the versatility of PANi–ZnO composites in detecting different analytes, as summarized in the following examples.

**Figure 9:**
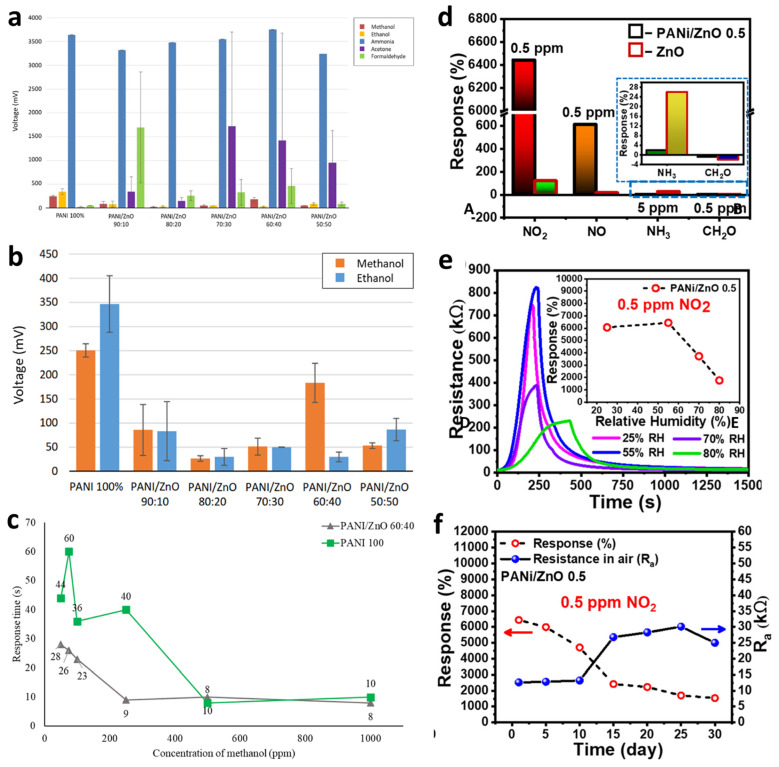
(**a**) Average responses of PANi and PANi/ZnO sensors toward different analytes. (**b**) Sensor responses of all samples to methanol and ethanol vapors. (**c**) Variation in response time with methanol concentration for PANi/ZnO (60:40) and pure PANi (100%) sensors. Reproduced from [212] with permission. (**d**–**f**) Gas responses of PANi/ZnO 0.5 and ZnO; time-dependent resistance at 25–80% RH (inset responses); 30-day R_a_ and response stability. Reproduced from [213] with permission.

In one study, PANi–ZnO nanocomposite sensors exhibited a remarkable response toward ammonia compared with other analytes such as acetone and formaldehyde (Figure 9a). The sensor operated in cycles of 1200 s exposure and 1200 s recovery, showing stable behavior over three repeated cycles. PANi and PANi/ZnO composites also showed excellent responses to methanol and ethanol vapors (Figure 9b). While pure PANi displayed higher sensitivity, it suffered from poor repeatability and structural degradation after multiple cycles due to alcohol-induced poisoning. In contrast, PANi/ZnO composites remained more stable, and higher ZnO loadings reduced the extent of degradation. The PANi/ZnO 60:40 sensor, for instance, demonstrated faster response times (8–10 s) at methanol concentrations between 200 and 1000 ppm, outperforming pure PANi, which required 40–50 s below 500 ppm (Figure 9c).

Figure 9d–f provide a comparative perspective on NO_2_ sensing using PANi/ZnO (0.5 wt%) and bare ZnO sensors. As seen in Figure 9d, the PANi/ZnO 0.5 sensor demonstrates significantly higher responses to 0.5 ppm NO_2_ than the ZnO-only counterpart, reinforcing the synergistic effect of the polymer in enhancing gas sensitivity. Figure 9e highlights the sensor’s performance under varying humidity levels (25–80% RH), where PANi’s hydrophobic nature appears to mitigate humidity-induced suppression of NO_2_ adsorption up to moderate RH levels (55%), although performance declines at higher humidity due to competitive water adsorption. Lastly, Figure 9f presents long-term performance trends, where PANi/ZnO 0.5 retains operational functionality over a 30-day period. While the response drops from 6440% to 1540%, the air resistance remains relatively stable after an initial adjustment phase, indicating satisfactory reproducibility and operational endurance. These studies collectively demonstrate that integrating ZnO into the PANi matrix enhances gas selectivity, accelerates response dynamics, and improves stability under both aging and environmental stressors positioning PANi–ZnO nanocomposites as effective candidates for room-temperature sensing of ammonia and nitrogen dioxide.

### 5.2. Gas Sensing Performance of Polypyrrole/ZnO Nanocomposites

Polypyrrole (PPy)–ZnO nanocomposites have been widely investigated as hybrid sensing materials owing to their synergistic combination of polymeric conductivity, ZnO’s chemical stability, and enhanced adsorption characteristics. The interaction between PPy and ZnO nanoparticles forms a p–n heterojunction, which significantly improves charge transfer efficiency and gas response under ambient conditions. Several reports have examined their performance toward oxidizing gases such as nitrogen dioxide (NO_2_), with particular focus on the influence of humidity and gas concentration.

**Figure 10:**
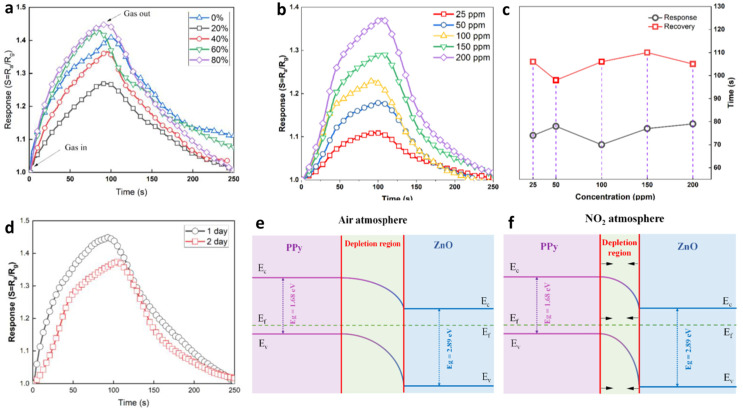
(**a**) Relationship between sensor sensitivity and relative humidity. (**b**) Gas response of ZnO/PPy nanocomposites and (**c**) corresponding response and recovery times at different NO_2_ concentrations. (**d**) Evaluation of sensor stability under repeated NO_2_ exposure. (**e**) Schematic energy-band diagram of ZnO/PPy nanocomposites in air and (**f**) under NO_2_ atmosphere [214].

In one representative study, the effect of relative humidity on NO_2_ sensing performance was evaluated at 200 ppm NO_2_ and various humidity levels (Figure 10a). At 0% relative humidity, the sensitivity (S) reached approximately 1.4089. As humidity increased to 20% and 40%, S decreased notably to around 1.364 and 1.268, respectively, due to competitive adsorption between NO_2_ molecules and water vapor on the sensor surface. Interestingly, at higher humidity (60–80%), the sensitivity increased again, reaching 1.428 and 1.447. This behavior was attributed to water-assisted adsorption: under low-temperature conditions, water molecules preferentially adsorb on ZnO-rich regions of the composite surface. When NO_2_ is introduced, it displaces these adsorbed water molecules, altering surface charge distribution and increasing sensitivity. After humidity testing, the sensor was stored in vacuum for 24 h to eliminate residual moisture.

Subsequent measurements explored the effect of NO_2_ concentration under 80% relative humidity (Figure 10b). The sensitivity increased consistently with NO_2_ concentration, showing values of 1.110, 1.179, 1.230, 1.292, and 1.374 for 25, 50, 100, 150, and 200 ppm, respectively. The dynamic response behavior is shown in Figure 10c, where the sensor exhibited significantly faster response times (≈20–30 s) compared to recovery times across all NO_2_ concentrations. Sensor stability was also assessed under repeated exposure conditions (Figure 10d). After one day of testing at 200 ppm NO_2_ and 80% RH, the maximum sensitivity decreased slightly from 1.445 to 1.374, while only minor variations in response and recovery times were observed, demonstrating good short-term reproducibility.

The underlying NO_2_ sensing mechanism is illustrated schematically in Figure 10e,f. Polypyrrole acts as a p-type semiconductor with its Fermi level (E_x_) positioned near the valence band, whereas ZnO nanoparticles are n-type with E_x_ close to the conduction band. Their intimate contact forms a p–n junction and corresponding depletion region (Figure 10e). Upon NO_2_ exposure, the overall electrical resistance decreases, confirming the p-type dominant behavior and PPy-mediated charge transport. Previous studies have shown that the interaction between NO_2_ molecules and the π-electron network of PPy results in charge transfer and a reduction in resistance. Consequently, the depletion region width narrows (Figure 10f), enhancing charge mobility and enabling the detection of even trace amounts of NO_2_.

## 6. Challenges and Future Perspectives

Despite the significant advancements in tuning ZnO’s gas sensing properties through dopants and polymer integration, several key challenges remain before these materials can be fully implemented in commercial sensor platforms. One of the most persistent issues is long-term stability, especially under real-world environmental conditions involving humidity, temperature fluctuations, and interfering gases. Over time, sensor surfaces may become passivated due to contaminant adsorption or polymer degradation, leading to signal drift and performance loss. Ensuring reproducibility across synthesis batches is another critical concern, particularly for sol–gel derived materials where small variations in parameters like pH, precursor concentration, or annealing temperature can drastically alter material properties. Moreover, selectivity continues to be a major hurdle, as many target gases share similar redox behaviors and adsorption kinetics, which can lead to cross-sensitivity and inaccurate readings. Designing sensors with integrated filtering layers or incorporating advanced data processing algorithms (e.g., machine learning) could offer practical solutions to these issues.

Looking ahead, the integration of ZnO-based composites into low-temperature and flexible sensing devices represents a promising and necessary evolution, especially in the context of wearable technologies, smart textiles, and Internet of Things (IoT) applications. The ability to operate efficiently at or near room temperature not only reduces energy consumption but also expands the potential for sensors to be embedded in portable or battery-powered systems. Polymer/ZnO composites, due to their inherent flexibility and tunability, are ideal candidates for such platforms. However, achieving reliable electrical contacts, mechanical durability under strain, and scalable fabrication methods for these flexible systems remains a technical challenge. Future work should also explore multi-functional and multi-gas sensing systems, potentially by incorporating additional sensing layers, functional coatings, or responsive polymers to create more intelligent and adaptive devices. Additionally, the synergy between light activation (e.g., UV-assisted sensing) and dopant/polymer strategies could further lower operating temperatures while enhancing sensitivity.

The data summarized in Table 1 highlights the diverse approaches to ZnO-based gas sensors, emphasizing the role of both transition and rare earth metal doping as well as polymer composites in enhancing sensor performance [191,192,193,212,213,214]. Notably, many polymer/ZnO nanocomposites exhibit extremely high sensitivity (e.g., >4000% response for NH_3_) and function effectively at room temperature, aligning well with the goals for low-power, flexible sensing. Meanwhile, rare-earth doped ZnO structures demonstrate impressive selectivity and fast response/recovery times for gases like NO_2_ and NH_3_. However, the variability in sensing temperatures, ranging from room temperature to 300 °C, underscores the ongoing need to develop materials that maintain high performance without thermal activation. This table serves as a strong testament to the tunability of ZnO-based sensors and the importance of carefully tailoring composition and structure to suit specific application needs.

**Table 1:** Effect of doping (transition metal and rare earth) and polymer integration on the sensing behaviors of ZnO.

Sample Name	Gas Name	Gas Conc.	Response	Response/Recovery Times	Sensing Temp.	Ref.
Doping by transition metal						
Fe-doped ZnO thick film	NH_3_	100 ppm	85% (resistance)	~50 s/~60 s	150 °C	[215]
Al-doped ZnO nanoparticles	CO	50 ppm	2.5 (S value)	30 s/45 s	200 °C	[216]
Ga-doped ZnO nanoparticles	CO_2_	100 ppm	1.8 (S value)	40 s/60 s	250 °C	[217]
CuO-ZnO composite	Acetaldehyde	50 ppm	18.2	23 s/36 s	200 °C	[218]
Cu-doped ZnO thin film	Propane (C_3_H_8_)	1000 ppm	~6 × 10^4^	Not specified	300 °C	[219]
Co-ZnO nanoflower (10% Co)	Isopropanol	5 ppm	12.2	330 s/475 s	225 °C	[220]
Mn-doped ZnO thin film	Ammonia (NH_3_)	200 ppm	23%	44 s/65 s	250 °C	[221]
Doping by Rare earth						
Gd-doped ZnO film	NH_3_	100 ppm	S = 18.2	39 s/11 s	Room Temp	[222]
La-doped ZnO film	H_2_S	100 ppm	S = 14.5	42 s/13 s	300 °C	[205]
La-doped ZnO film	CO_2_	500 ppm	S = 3.2	29 s/17 s	Room Temp	[205]
ZnO-150 (Ce-doped)	NO_2_	Not specified	132.44%	231.7 s/732.5 s	Room temperature	[223]
Dy-doped ZnO film	NO_2_	1 ppm	S = 10.3	35 s/15 s	Room Temp	[224]
Nanocomposites with conductive polymer						
ZnO-PANI nanocomposite	NH_3_	100–500 ppm	Sensitivity increases with ZnO wt% (max at 6 wt%)	10–30 s/up to 1200 s (20 min)	Room Temp	[225]
PANI/nano-ZnO FET sensor	H_2_	100 ppm	Enhanced vs pure PANI	Not specified	Room Temp	[226]
PANi/ZnO NR composite	NH_3_	0.05/2.5 ppm	130% (0.05 ppm)/20,920% (2.5 ppm)	Not specified	Room Temp	[213]
ZnO/PANI composite	Ethanol	100 ppm	S = 20	~20 s	Room Temp	[227]
Chitosan-PEG/ZnO composite	Acetone	0.5–5 ppm	LOD ≤ 0.96 ppb; linear and selective	~5 min exposure/recovery	~29 °C	[228]
ZnO/PANI nanocomposite	NH_3_	100 ppm	4300%	Not specified	Room Temp	[229]

## 7. Conclusions

In this comprehensive review, we have explored advancements in enhancing the gas sensing properties of sol–gel-derived ZnO through dopant engineering and polymer integration. The modifications introduced by various dopants, including transition metals (e.g., Co, Ni, Cu) and rare-earth elements (e.g., La, Gd), have demonstrated significant improvements in the sensitivity, selectivity, and operational stability of ZnO-based gas sensors. These dopants influence the electronic structure, defect density, and surface reactivity of ZnO, thereby optimizing its interaction with target gases such as H_2_, CO, CH_4_, NH_3_, and NO_2_. Additionally, the incorporation of polymers like polyaniline (PANI) and polypyrrole (PPy) into ZnO matrices has been shown to further enhance gas sensing performance, particularly at room temperature. These polymer composites provide functional groups for selective gas interactions, improve mechanical flexibility, and facilitate efficient charge transfer at the ZnO–polymer interface. Despite these advancements, challenges such as long-term stability, reproducibility, and selectivity in complex environments remain. Future research should focus on addressing these issues through the development of multi-functional and multi-gas sensing systems, integration of advanced data processing algorithms, and exploration of light-assisted detection methods. Comparative tagging in this review shows that sol–gel routes systematically tune defect chemistry and microstructure toward improved sensitivity and lower operating temperature, especially in doped and PANi/PPy hybrid systems. Overall, the synergistic effects of dopant engineering and polymer integration present a promising pathway for the rational design of high-performance ZnO-based hybrid sensors, paving the way for their application in environmental monitoring, industrial safety, and biomedical diagnostics.

## Data Availability

The data that support the findings of this study are available from the corresponding author upon reasonable request.
